# An Adaptive Deghosting Method in Neural Network-Based Infrared Detectors Nonuniformity Correction

**DOI:** 10.3390/s18010211

**Published:** 2018-01-13

**Authors:** Yiyang Li, Weiqi Jin, Jin Zhu, Xu Zhang, Shuo Li

**Affiliations:** School of Optoelectronics, Beijing Institute of Technology, Key Laboratory of Photo-electronic Imaging Technology and System, Ministry of Education of China, Beijing 100081, China; 20130263@bit.edu.cn (Y.L.); zhujin6319@126.com (J.Z.); 3120140264@bit.edu.cn (X.Z.); 3120140238@bit.edu.cn (S.L.)

**Keywords:** fixed pattern noise, nonuniformity correction, noise estimation, neural network

## Abstract

The problems of the neural network-based nonuniformity correction algorithm for infrared focal plane arrays mainly concern slow convergence speed and ghosting artifacts. In general, the more stringent the inhibition of ghosting, the slower the convergence speed. The factors that affect these two problems are the estimated desired image and the learning rate. In this paper, we propose a learning rate rule that combines adaptive threshold edge detection and a temporal gate. Through the noise estimation algorithm, the adaptive spatial threshold is related to the residual nonuniformity noise in the corrected image. The proposed learning rate is used to effectively and stably suppress ghosting artifacts without slowing down the convergence speed. The performance of the proposed technique was thoroughly studied with infrared image sequences with both simulated nonuniformity and real nonuniformity. The results show that the deghosting performance of the proposed method is superior to that of other neural network-based nonuniformity correction algorithms and that the convergence speed is equivalent to the tested deghosting methods.

## 1. Introduction

Imaging systems based on infrared focal plane arrays (IRFPAs) have been widely used in military and civilian applications. However, as they are limited by the manufacturing quality and the level of the processing technology, the responsiveness of the individual photodetectors in the focal plane array will vary from detector to detector. This is known as the nonuniformity of an IRFPA and is also called fixed pattern noise (FPN). Nonuniformity severely corrupts infrared images and must be corrected in practical applications. To this end, many nonuniformity correction (NUC) techniques have been proposed to compensate for FPN. These NUC techniques are categorized into two classes, namely, calibration-based and scene-based techniques.

Calibration-based nonuniformity correction (CBNUC) algorithms are generally used to reduce FPN, with the simplest and most used one being the two-point calibration [1]. However, as FPN drifts temporally, CBNUC algorithms must be repeated by inserting a uniform radiation source into the view, thus affecting the imaging in use [2]. To address this disadvantage, scene-based NUC (SBNUC) algorithms were proposed. SBNUC algorithms use scenes to fix the FPN time fluctuation without a uniform radiation source. These approaches are divided into neural network (NN) algorithms [3], temporal high-pass algorithms [4,5], constant–statistical algorithms [6], and registration-based algorithms [7,8]. Among them, the NN-based SBNUC algorithms have received extensive attention due to the few requirements of computing resources and storage spaces, and this type of correction is very likely to replace CBNUC in the infrared imager.

**Figure 1:**
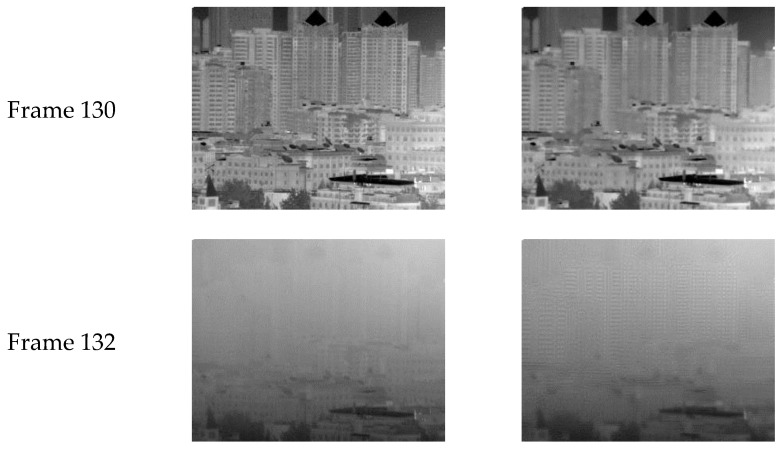
An example infrared sequence processed by Scribner’s method. The first column is the original image, the second column is the processed image.

The NN-based SBNUC algorithm was first proposed by Scribner in [3]. This algorithm estimates the needed gain and offset parameters on a frame-by-frame basis by means of the least mean square (LMS) method. However, this method does not handle the problem of ghosting artifacts, which is a collateral effect caused by the NUC on the scene. As shown in Figure 1, the first row is frame 130 and the scene has remained stationary for 130 frames. By using Scribner et al.’s algorithm to reduce the FPN, the textures of the building become blurred. The ghosting artifacts appear when the hand blocks the field of view (FOV) in frame 132. Such artifacts have become an obstacle to the application of the algorithm.

In order to reduce ghosting artifacts, Vera et al. improved Scribner et al.’s retina-like neural network approach method using an adaptive learning rate [9]. Rossi et al. pointed out that ghosting artifacts are generated mainly in strong edge areas. Hence, instead of classical low-pass filters, they introduced a bilateral filter which preserves edges [10]. Zhang et al. introduced an edge detection mechanism in the calibration process [11]. The binarized edge detection results are used to control the learning rate. As with a bilateral filter, the threshold used to distinguish edges and noise in this algorithm is difficult to select. These two methods consistently highlight residual nonuniformity noise, which is considered to be false edges. Torres et al. improved the learning rate function, following temporal variations in the operation point of each detector [12]. However, the frame motion detection is based on phase correlation. Increasing the step size in the local motion blurs stationary areas. Hardie et al. proposed a deghosting method based on a temporal gate [13]. By gating the update of the NUC parameters, this method avoids the accumulation of incident radiation estimation errors during long motion pauses. Up to now, Hardie et al.’s deghosting strategy has been widely used [14]. Nonetheless, for some common scenes, such as when the imager is observing a scene with jitter, Hardie et al.’s deghosting algorithm fails. Fan et al. proposed a combined temporal and spatial deghosting method to compensate for the deghosting failure of Hardie et al.’s algorithm [15]. However, this method’s spatial limits with a hard threshold are similar to those of the previous method. In cases where the nonuniformity noise is serious, the constant spatial threshold makes it difficult to take into account noise removal and the ghosting inhibition.

This paper proposes a combined adaptive spatial and temporal deghosting (AST) method. We use adaptive threshold edge detection so that the rigidity of the spatial limitation can be adjusted according to the residual nonuniformity noise. In other words, when the noise is serious, a large threshold is used to enhance the de-noising ability. In the opposite case, the threshold will become smaller to suppress the ghosting artifacts.

The rest of the paper is organized as follows. Related work is introduced in Section 2. Section 3 is dedicated to a detailed description of the proposed algorithm, and also highlights the improvements made. In Section 4, the NUC experimental results are discussed. In Section 5, the efficiency of the algorithm is analyzed. The conclusion is presented in Section 6.

## 2. Neural Network-Based Nonuniformity Correction

### 2.1. Nonuniformity Observation Model

We begin by assuming that each infrared detector is characterized by a linear model, without considering the additive temporal noise. For the (*i*,*j*)th detector in the IRFPA, the measured readout signal *y_i,j_* at a given time *n* can therefore be expressed as:

(1) $$y_{i,j}\left(n\right)=G_{i,j}\left(n\right)x_{i,j}\left(n\right)+O_{i,j}\left(n\right),$$

where *G_i_*_,*j*_ (*n*) and *O_i_*_,*j*_ (*n*) are the gain and the offset coefficients of the (*i*,*j*)th detector, and *x_i_*_,*j*_ (*n*) is the real incident infrared photon flux collected by the respective detector.

The main purpose of the NUC method is to estimate the value of *x_i_*_,*j*_ (*n*) accurately. Using Equation (1), the value of *x_i_*_,*j*_ (*n*) can be obtained by the following equation:

(2) $$x_{i,j}\left(n\right)=w_{i,j}\left(n\right)y_{i,j}\left(n\right)+b_{i,j}\left(n\right),$$

where the new parameters *w_i,j_* (*n*) and *b_i,j_* (*n*) are related to the gain and offset coefficients, respectively, of each detector as follows:

(3) $$w_{i,j}\left(n\right)=\frac{1}{G_{i,j}\left(n\right)},\;b_{i,j}\left(n\right)=\frac{O_{i,j}\left(n\right)}{G_{i,j}\left(n\right)},$$

### 2.2. Deghosting Methods

The key of the SBNUC algorithm is to obtain multiple sets of incident values and their corresponding response readout signals to calculate the gain and offset correction parameters. In the NN-based SBNUC algorithm, the estimated incident radiation value is calculated as:

(4) $${\hat{X}}_{i,j}\left(n\right)=\kappa \left(x_{i,j}\left(n\right)\right),$$

where the spatial low-pass filter *κ* constitutes the hidden layer.

Commonly used filters are the four-neighborhood filter, the Gaussian filter, the bilateral filter, the guide filter, and the non-local average filter [16]. These filters are used to force the estimated desired image closer to the real incident image. However, any estimation of the incident radiation cannot be accurate, and, even worse, this estimation is, in most cases, wrong. Therefore, the NN algorithms do not directly calculate the gain and offset matrix according to the estimated value $\hat{X}$ and the readout signal *y*. However, according to the LMS algorithm, the gain and offset correction parameters are constantly iterated by gradient descent through a large amount of radiation estimation values $\hat{X}$ and the gray values *y*. This also improves the dynamic range of correction. Therefore, the final error function is given by:

(5) $$E_{i,j}\left(n\right)={\left(w_{i,j}\left(n\right)y_{i,j}\left(n\right)+b_{i,j}\left(n\right)-{\hat{X}}_{i,j}\left(n\right)\right)}^{2},$$

Using the LMS algorithm, the correction parameters *w_i,j_* and *b_i,j_* are recursively and smoothly updated with a portion of each respective error gradient, as follows:

(6) $$w_{i,j}\left(n+1\right)=w_{i,j}\left(n\right)-\varepsilon_{i,j}\left(n\right)\left(x_{i,j}\left(n\right)-{\hat{X}}_{i,j}\left(n\right)\right)y_{i,j}\left(n\right),$$

(7) $$b_{i,j}\left(n+1\right)=b_{i,j}\left(n\right)-\varepsilon_{i,j}\left(n\right)\left(x_{i,j}\left(n\right)-{\hat{X}}_{i,j}\left(n\right)\right),$$

where *ε* is the step size known as learning rate, which controls the convergence speed of the algorithm. Higher values of *ε* can provide a faster convergence, but the results are unreliable. Conversely, smaller values of *ε* can produce more reliable results, but with a lower rate of convergence. A suitable value for learning rate ε can better mitigate ghosting artifacts. Vera et al. use local variance to control *ε*. A large local variance means that the incident radiation estimation error is large, so a small ε is used. A large ε is used as a contrast. This method reduces ghosting artifacts to a certain extent and actually increases convergence speed. However, because the learning rate is never actually set to zero with the adaptive LMS algorithm, this method does not eliminate burn-in ghosting for long motion pauses. The ghosting artifacts occur when motion resumes.

Hardie et al. use a temporal threshold to gate the step size. With a stationary scene, the pixel gray value does not change significantly from one frame to the next. Therefore, a threshold can be set to stop NUC parameters from being updated. The gating method can be represented by:

(8) $$\varepsilon_{i,j}\left(n\right)=\{\begin{array}{cc} \frac{k_{alr}}{1+\sigma_{y_{i,j}\left(n\right)}} & |{\hat{X}}_{i,j}\left(n\right)-Z_{i,j}\left(n\right)|>T\\ 0 & \mathrm{else} \end{array},$$

(9) $$Z_{i,j}\left(n+1\right)=\{\begin{array}{cc} {\hat{X}}_{i,j}\left(n\right) & |{\hat{X}}_{i,j}\left(n\right)-Z_{i,j}\left(n\right)|>T\\ Z_{i,j}\left(n\right) & \mathrm{else} \end{array},$$

where *σ_y_* is the local spatial standard deviation of the input image *y*. The parameter *k_alr_* is the maximum step size. *T* is the temporal threshold, and *Z* is used to detect the temporal motion.

The scene information has not been taken into account in Hardie et al.’s method. When the edges appear and disappear on the same pixel, or when the image scene shakes, ghosting artifacts can still be generated. Fan et al. proposed a combined temporal and spatial deghosting method to solve the problem.

(10) $$\varepsilon_{i,j}\left(n\right)=\{\begin{array}{cc} \frac{k_{alr}}{1+\sigma_{y_{i,j}\left(n\right)}} & \left|{\hat{X}}_{i,j}\left(n\right)-Z_{i,j}\left(n\right)\right|>T\;AND\;S_{i,j}\left(n\right)=1\\ 0 & \mathrm{else} \end{array},$$

(11) $$Z_{i,j}\left(n+1\right)=\{\begin{array}{cc} {\hat{X}}_{i,j}\left(n\right) & |{\hat{X}}_{i,j}\left(n\right)-Z_{i,j}\left(n\right)|>T\;AND\;S_{i,j}\left(n\right)=1\\ Z_{i,j}\left(n\right) & \mathrm{else} \end{array},$$

(12) $$S_{i,j}\left(n\right)=\{\begin{array}{cc} 1 & \forall (p,q)\in \Omega ,\left|{\hat{X}}_{p,q}\left(n\right)-{\hat{X}}_{i,j}\left(n\right)\right|\leq T_{s}\\ 0 & \mathrm{else} \end{array},$$

where *S_i,j_* (*n*) is the spatial local correlation. *S_i,j_* (*n*) = 1 means that the pixel (*i*,*j*) may be in the uniform area. Ω is the local window.

One can see here that the correction parameters can only be updated if a pixel satisfies both the temporal motion detection and the spatial correlation detection. This method can, for some cases, effectively compensate for the deghosting failure of Hardie et al.’s algorithm. However, the spatial threshold of the algorithm is constant. When the noise is serious, the calculated local spatial correlation is large, and it is necessary to use a large spatial threshold to ensure the removal of nonuniformity noise. With the gradual lowering of nonuniformity noise through the iteration correction, the local spatial correlation of the image is mainly affected by the scene. The previously determined large spatial threshold cannot continue to limit the correction parameters that are updating at the edges, so ghosting artifacts can also be generated. Therefore, a more reasonable threshold should be adaptively adjusted according to residual nonuniformity noise.

## 3. Proposed Deghosting Method

### 3.1. Combined Temporal Gate and Edge Detection

**Figure 2:**
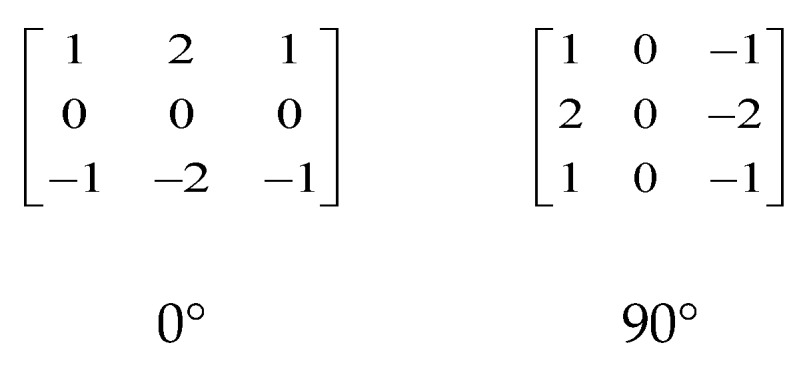
Sobel templates in two directions.

Generally, a pixel located in the flat area has a low estimated error of incident radiation, so it should not be limited for correction. In contrast, the NUC parameter updating in the area with scene textures should be stopped, as the error of the incident radiation estimation would be large [11,12,15]. As the scene textures are often directional, we used the Sobel operator, as shown in Figure 2, to calculate the image gradient in horizontal and vertical directions in order to locate the scene information areas in the image.

Here, the object of the edge detection is the low-noise corrected image. Subsequently, we define the gradient image as follows:

(13) $$edge_{x_{i,j}}=\left|e_{0}\right|+\left|e_{90}\right|,$$

**Figure 3:**
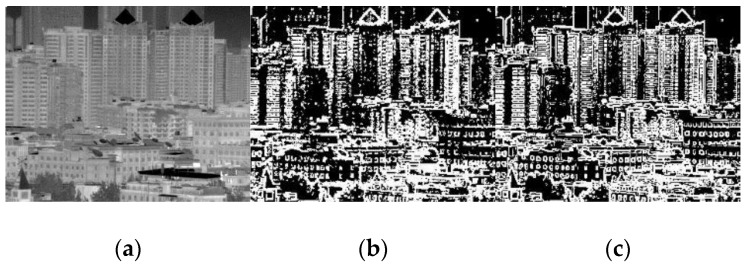
Comparison of image edge extraction: (**a**) original image; (**b**) spatial correlation by 3 × 3 window; and (**c**) Sobel operator.

In the obtained gradient image, the pixels with larger gray values exhibit a notably higher error in estimating the incident radiation in the hidden layer, and vice versa. The purpose of edge detection in our algorithm is therefore to extract the large gradients that are mainly caused by the scene textures. The spatial threshold *S* is used to binarize the *edge_x_* to obtain the large gradient pixel set denoted as Φ. Compared with Fan et al.’s spatial correlation operator, the Sobel operator, based on the first order gradient, is less sensitive to the shot noise, as shown in Figure 3b,c. In Figure 3, the two operators used the same window size (3 × 3) and threshold (20). Here, a more intelligent edge detection operator such as Canny was not used because Sobel preserves the most primitive gradient information of the image and has a relatively smaller computational complexity.

In summary, the combined temporal gate and edge detection controlled learning rate can be expressed as:

(14) $$\varepsilon_{i,j}\left(n\right)=\{\begin{array}{cc} \frac{k_{alr}}{1+\sigma_{y_{i,j}\left(n\right)}} & |{\hat{X}}_{i,j}\left(n\right)-Z_{i,j}\left(n\right)|>T\;AND\;x_{i,j}\left(n\right)\notin \Phi \\ 0 & \mathrm{else} \end{array},$$

(15) $$Z_{i,j}\left(n+1\right)=\{\begin{array}{cc} {\hat{X}}_{i,j}\left(n\right) & |{\hat{X}}_{i,j}\left(n\right)-Z_{i,j}\left(n\right)|>T\\ Z_{i,j}\left(n\right) & \mathrm{else} \end{array},$$

Consequently, the correction parameters can be updated only if a pixel responds to the temporal motion detection and is located in the non-edge region. The key to this method lies in how to obtain an intelligent threshold *S* that is associated with the nonuniformity noise gradient.

### 3.2. Adaptive Spatial Threshold

Because of the influence of FPN, it is difficult to accurately diagnose whether the image gradient is caused by scene or noise. When the spatial threshold is too large, it is not possible to limit the correction parameters that are updating in the edge area, and this may in turn cause ghosting artifacts. On the other hand, a threshold that is too small causes the presence of noise that cannot be removed. For this reason, we divided the correction process into the denoising stage and the deghosting stage. (1) During the denoising stage, the image gradient is greatly affected by the nonuniformity noise, and the main task of the algorithm is to remove such noise. At this point, the update of the correction parameters should be as unlimited as possible. A large adaptive spatial threshold should therefore be used; (2) After hundreds of iterations, when the noise has been gradually removed, the algorithm should focus on suppressing ghosting. Here, a smaller, adaptive spatial threshold should be used to conserve the detail gradients. A flag *STA* is used to represent the two states. *STA* = 1 denotes the denoising stage, otherwise it is the deghosting stage.

Regarding the frame-by-frame iteration correction algorithm, the more noise the correction matrix contains, the smaller the residual nonuniformity in the corrected image. Without loss of generality, it is assumed that the gain nonuniformity is either a known constant given by the infrared imager manufacturer, or that it can be initially calculated by using a priori information on the particular infrared imager under study [2,17,18]. Thus, we only need to correct the offset nonuniformity that results from the temperature drift. In the denoising stage, the noise contained in the offset correction matrix is continuously increased. At the same time, the FPN in the corrected image gradually decreases. After multiple frame iterations, the noise in the correction matrix remains essentially unchanged, and the dynamic correction is temporarily completed and then enters the deghosting stage. Thus, the temporal variation of the first order gradient of the correction matrix can be used as the basis for the convergence of the correction. The Sobel operator is used to mask the correction matrix, and *edge_b_* is obtained. The mean value of *edge_b_* is denoted as $\bar{{\mathrm{edge}}_{b}}$. The larger $\bar{{\mathrm{edge}}_{b}}$ is, the more noise is contained in the correction matrix, which means that less noise is included in the corrected image. The temporal variation of $\bar{{\mathrm{edge}}_{b}}$ is represented as:

(16) $$\sigma_{i,j}^{T}\left(n\right)=\max\left({\bar{edge}}_{b\left(n\right)},{\bar{edge}}_{b\left(n-1\right)},\ldots ,{\bar{edge}}_{b\left(n-m+1\right)}\right)-\min\left({\bar{edge}}_{b\left(n\right)},{\bar{edge}}_{b\left(n-1\right)},\ldots ,{\bar{edge}}_{b\left(n-m+1\right)}\right),$$

where *m* is the temporal parameter. Usually, when $\sigma_{i,j}^{T}(n)$ is large, the gradient of the correction matrix changes greatly, which indicates that the correction is not convergent and that the residual noise of the corrected image is serious. On the contrary, when the correction process tends to be stable, the corrected image becomes noiseless. Thus, it is possible to distinguish the stages of denoising and deghosting.

(17) $$\sigma_{i,j}^{T}\left(n\right)=STA(n)=\{\begin{array}{cc} 1 & \sigma_{i,j}^{T}\left(n\right)>T_{1}\\ 0 & \sigma_{i,j}^{T}\left(n\right)\leq T_{1} \end{array},$$

where *T*_1_ is the threshold. In the application, we selected *T*_1_ = 1 and *m* = 50 to achieve satisfactory results.

According to a previous analysis, the design of the edge detection threshold is determined by the severity of nonuniformity noise. In the two correction stages that have been distinguished, the threshold has different strategies. Inspired by the successful application of noise estimation algorithms in the field of computer vision, we used some of the noise evaluation ideas found in the literature [19,20,21,22]. For images with offset noise, the degradation model can be represented as:

(18) $$edge_{g_{i,j}}=edge_{f_{i,j}}+edge_{O_{i,j}},$$

where *f* is the noiseless image, and *g* is the corrupted image. Without loss of generality, we assume that images always contain some flat areas, such as roads, the sky, and so on. In these areas, there is often some noise distribution but almost no scene texture. Thus, Equation (18) can be rewritten as:

(19) $$edge_{g_{i,j}}=edge_{O_{i,j}},$$

That is, the gradient of nonuniformity noise is the same as that of the corrected image in the flat area. The flat area in the image is located by calculating the local variance *V* of each *W* × *W* window; the region that has the least local variance is the flat region. The formula is as follows:

(20) $$(Np,Nq)=loc\left(\underset{i,j}{\min}\left\{{\underset{W\times W}{V}}_{g_{i,j}}\right\}\right),$$

The *W* × *W* area with (*Np*, *Nq*) at its the center is the flattest. The maximum gradient of this area can be used as the gradient level of noise, just as follows:

(21) $$nu_{g}=\max\left\{edge_{g_{i,j}},i,j\in \Theta \right\},$$

where *nu_g_* represents the noise gradient of the image *g* and Θ represents the set of pixels in the flattest area. In this way, we have estimated the noise gradient using an image. However, due to the global distribution of nonuniformity noise, the noise gradient calculated via the flattest region is always too small. Therefore, in the denoising stage, in order to fully remove the FPN, an expansion coefficient *e* is used to adjust the estimated noise gradient, and the result serves as the adaptive edge detection threshold for this stage. In the deghosting stage, the noise gradient estimated via the corrected image is used directly as the threshold. As a result, the adaptive edge detection threshold *S* can be represented as:

(22) $$S\left(n\right)=\{\begin{array}{cc} e\cdot nu_{x\left(n\right)} & STA\left(n\right)=1\\ nu_{x\left(n\right)} & STA\left(n\right)=0 \end{array},$$

We can see that the larger the value of *e*, the more aggressive the noise removal ability, but the weaker the deghosting ability. We recommended that 2 ≤ *e* ≤ 10.

### 3.3. Algorithm Summary

**Figure 4:**
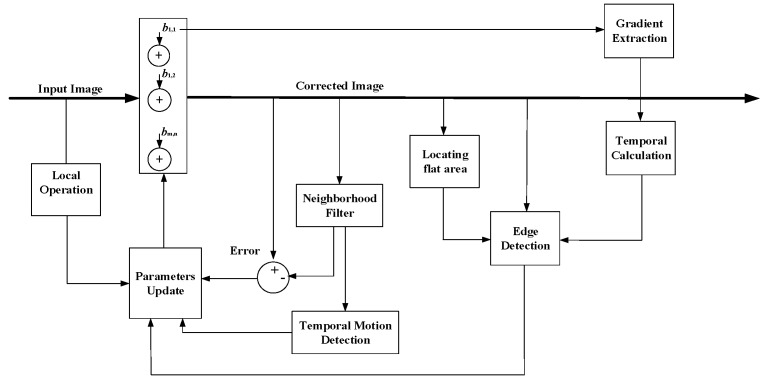
Scheme of proposed adaptive spatial and temporal deghosting–least mean square nonuniformity correction (AST–LMS NUC) algorithm.

An overview of our algorithm is presented in Figure 4. The input image is the readout data *y_i_*_,*j*_, which is processed by the offset NUC parameter to get the corrected image *x_i,j_*. The neighborhood filter is applied to the corrected image *x_i,j_*, generating the target ${\hat{X}}_{i,j}$ in order to calculate the error function *E_i_*_,*j*_. Edge detection extracts the large gradient of *x_i,j_*, and is combined with temporal motion detection and local operation to control the step factor of the parameter updates. Following this, the steepest descent method is used to obtain the NUC parameters for the next frame. Gradient extraction calculates the gradients of the offset NUC matrix to obtain $\bar{{\mathrm{edge}}_{b}}$, and then *STA* is obtained through a temporal calculation. The result of locating the flat area modulated by the *STA* is used as the final edge detection threshold. Comparing with the block schemes in [9,15], the adaptive threshold calculation composed of the above three modules is our algorithm’s main contribution.

## 4. Experimental Results

The main goal of this section is to evaluate the efficacy of the proposed adaptive algorithm. The algorithm was tested using an image sequence with simulated nonuniformity and two real infrared image sequences.

### 4.1. Nonuniformity Correction with Simulated Data

A clean 14 bit infrared video sequence was acquired by a 336 × 256 focal-plane array (FPA) uncooled imager (FLIR TAU2-336). The corrupted video sequences were obtained using a global synthetic offset with a zero-mean Gaussian distribution with a standard deviation of 30 and a vertical stripe offset with a standard deviation of 15. The entire acquisition was performed outdoors. Scenes typically known to easily cause ghosting artifacts were imaged to verify the ability of the adaptive threshold to remove noise and suppress ghosting. For the first 600 frames, the imager moved in a random and consistent manner. Then the imager jittered toward the buildings. The imager suddenly moved away from the FOV at frame 955. In total, there were 1000 frames.

The metric used to measure the NUC performance was the peak signal-to-noise ratio (PSNR), which is widely used in the image processing literature to quantify the differences between two images, and it is defined as:

(23) $$RMSE=\sqrt{\frac{1}{MN}\sum_{i,j}{\left(I_{i,j}-{\hat{I}}_{i,j}\right)}^{2}}$$

(24) $$PSNR=20\cdot \log_{10}\left(\frac{2^{c}-1}{RMSE}\right)$$

where *c* is the number of bits of image data (here *c* = 14). *I_i,j_* is the clean raw image and ${\hat{I}}_{i,j}$ is the corrected image.

In the experiment, we used Hardie et al.’s Gated–least mean square (Gated–LMS) algorithm [13], Fan et al.’s combined spatial and temporal deghosting–least mean square (ST–LMS) algorithm [15] and the algorithm proposed in this paper to calibrate the offset parameters of the image sequence. All of the LMS methods used the same *k_al_* = 0.8, *T* = 8 and a four neighborhood filter. The spatial correlation window used in ST–LMS was 7 × 7, and the spatial threshold *T_s_* was set to 50 in order to ensure the removal of the noise. Our algorithm used *W* = 7 to calculate the threshold, and *e* was set to 8.

**Figure 5:**
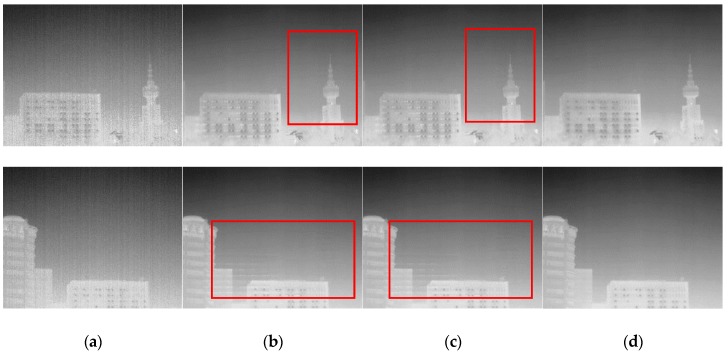
Typical scenes in the correction process: (**a**) uncorrected image; (**b**) Gated–least mean square (Gated-LMS); (**c**) combined spatial and temporal deghosting–least mean square(ST-LMS); (**d**) our image. The top row is the 954th frame, when the imager jittered toward the outdoor scene. The bottom row is the 959th frame when the imager suddenly move.

Figure 5 shows some typical images in the sequence. The top row in Figure 5 is the 964th frame when the infrared imager was jittering against the outdoor scene. The 959th frame, after the FOV suddenly changed is shown in the bottom row in Figure 5. One can see that the FPN was almost removed after the processing of the three algorithms, but the Gated–LMS and ST–LMS have obvious ghosting artifacts, as indicated in the red boxes in Figure 5b,c. There were almost no ghosting artifacts in the image processed by our algorithm, as shown in Figure 5d. The visual effect of our algorithm is more satisfactory than the two compared algorithms, and this can be more comprehensively seen in Video S1.

**Figure 6:**
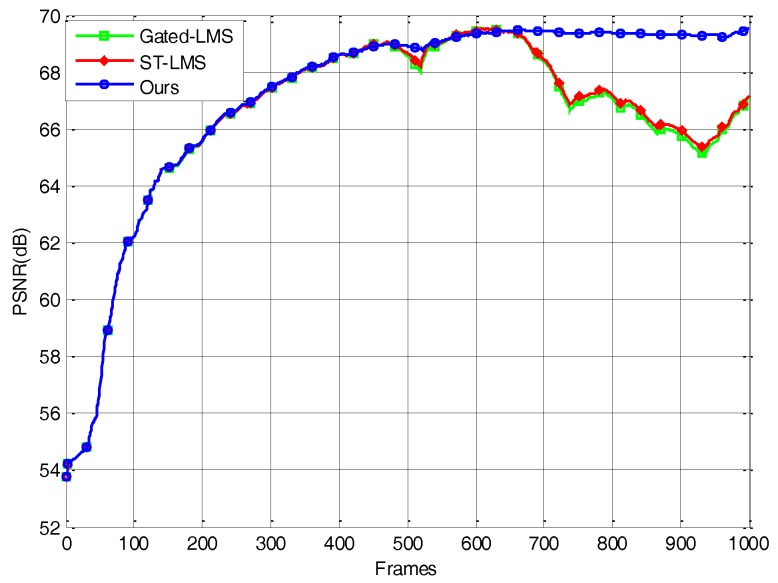
PSNR versus frame number for the tested algorithms using simulated nonuniformity data.

The PSNR curve is shown in Figure 6. As can be seen, in the first 480 frames, the PSNR curves of the three algorithms coincide with each other and rise rapidly due to the random motion of the imager. This shows that the adaptive threshold in our algorithm did not hinder the removal of nonuniformity noise and that the convergence speed was as fast as that of Gated–LMS’s. Subsequently, due to the unintentional jitter accompanying the movement of the imager, the edges appeared and disappeared at the same position. The PSNR curves of the Gated–LMS and ST–LMS show obvious downward spikes caused by ghosting artifacts in the image. The imager was jittered deliberately from frame 600 onwards. At this point, the PSNR curves of the Gated–LMS and ST–LMS remain in a downward trend, and there are multiple downward spikes. At this stage, although the spatial threshold of the ST–LMS failed to suppress most ghosting artifacts, it also stopped the iterative correction of some strong edges, so that the PSNR curve is slightly higher than that of the Gated–LMS. During the jitter process, the PSNR of our algorithm stays at the highest level reached, which is obviously higher than that of the other two algorithms.

**Figure 7:**
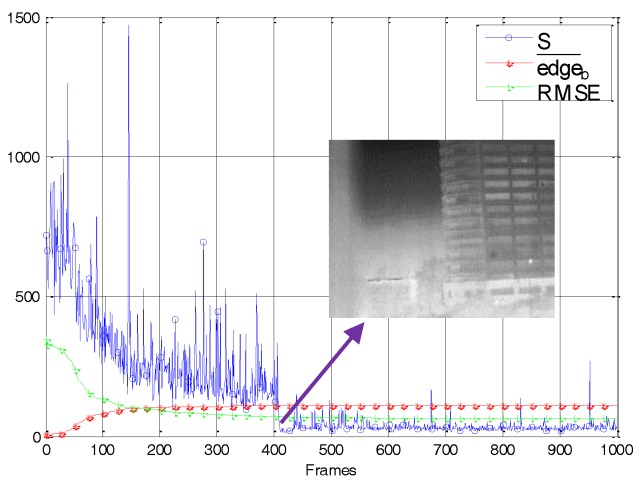
The variation of the important variables in our algorithm during the simulated nonuniformity data calibration.

In addition, as shown in Figure 7, we analyzed the frame-by-frame variation of the correction matrix gradient $\bar{{\mathrm{edge}}_{b}}$ and the adaptive threshold *S*. The RMSE was used as an index of residual nonuniformity noise. Specifically, lower RMSE values indicate less residual nonuniformity noise, and vice versa. In Figure 7, in order to facilitate the observation of the RMSE curve, we had expanded its value by 10 times. The figure shows that in the first 200 frames, due to serious noise in the image, there were always some correction parameters that were updating, regardless of whether the imager was moving or stationary. Until frame 425, the correction matrix was close to obtaining the noise distribution of the original image. Its gradient no longer increased significantly. At this point, the corresponding RMSE was reduced to 4.8, and the residual nonuniformity noise of the corrected image was weak, as indicated by the arrow in Figure 7. After frame 425, both the $\bar{{\mathrm{edge}}_{b}}$ curve and the RMSE curve remain stable. In other words, when the $\bar{{\mathrm{edge}}_{b}}$ changed violently, the residual noise was strong. On the contrary, when $\bar{{\mathrm{edge}}_{b}}$ reached stability, the residual noise was weak. The curve shows that the adaptive spatial threshold *S* always shows some fluctuations influenced by the scene information. In general, however, it exhibits a downward state. The *S* curve decreases sharply after 425 frames, which is consistent with the RMSE curve. After frame 425, because of the regulation of $\bar{{\mathrm{edge}}_{b}}$, the *S* curve fluctuates near 20, ensuring that the ghosting artifacts were suppressed while some weak noise was removed. The analysis results show that the index $\bar{{\mathrm{edge}}_{b}}$ used in the algorithm can effectively characterize the intensity of residual nonuniformity noise in the corrected image. Under its control, the adaptive spatial threshold is able to restrict the ghosting artifacts more intelligently without affecting the convergence speed of the algorithm.

### 4.2. Nonuniformity Correction with Real Data

Because it is difficult to use PSNR as the NUC evaluation index for real data, and because the FPN is expressed as spatial high frequency information, the amount of FPN present in a real image is usually measured using the roughness index.

(25) $$\rho =\frac{{\left\|h_{1}*I\right\|}_{1}+{\left\|h_{2}*I\right\|}_{1}}{{\left\|I\right\|}_{1}},$$

where *h*_1_ and *h*_2_ are a horizontal and vertical difference filter, respectively; *I* is the image under analysis; and ${\left\|f\right\|}_{1}$ is the *l*^1^ norm of *f*. Specifically, the lower the value for *ρ*, the lower the FPN, and vice versa.

The first real infrared data was collected for the indoor scene at 4 p.m., using a 320 × 240 InSb FPA cooled infrared imager from Cedip. The imager operates in the 3−5 μm range and works at 50 frames per second (FPS). The F-number of the optics is 0.75. The 14-bits image data of the detector was collected with only a gain nonuniformity calibration-based correction. The imager was moved continuously in the first 540 frames. Subsequently, while it was still facing the complex indoor scene, the FOV was repeatedly blocked by hand. In total, there were 2000 frames.

The selected algorithms were used to correct the offset parameters of the video sequence. All LMS algorithms used *k_al_* = 2, *T* = 10, and a four neighborhood filter. As the FPN was serious and the signal was almost submerged by noise, the ST–LMS used *T_s_* = 80 and a 7 × 7 spatial correlation window. The parameters in our algorithm were the same as those for the simulated experiment. This experiment mainly tested the convergence speed and the deghosting method of the tested algorithms for real nonuniformity noise.

**Figure 8:**
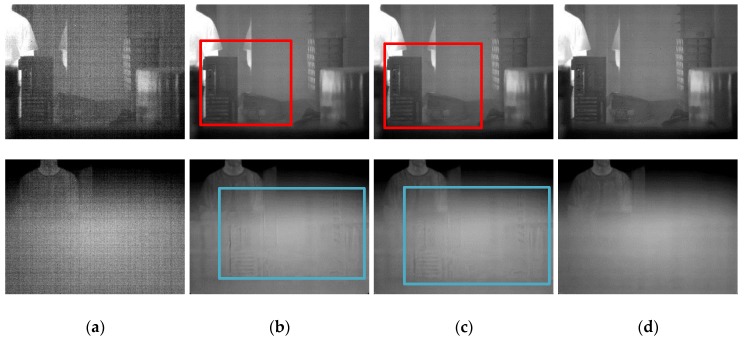
NUC performance comparison of the real nonuniformity infrared sequence: (**a**) un-corrected image; (**b**) Gated–LMS; (**c**) ST–LMS; (**d**) our image. The top row is the 906th frame, when the imager jittered toward the indoor scene. The bottom row is the 1965th frame, when the FOV was blocked by a hand.

Video S2 compares the tested algorithms for a visual evaluation. It is apparent that our algorithm effectively generated fewer ghosting artifacts than the other algorithms. When the imager remains stationary due to the platform’s weak jitter, the temporal variations of grayscale in the same pixel will be higher than the threshold. In this case, the Gated–LMS blurs the details regardless of the intensity of the gradient, just as shown in the red box in the top row of Figure 8b. The spatial threshold of ST–LMS retains some textures, but the details within the threshold are gradually degraded, as shown in the red box in the top row of Figure 8c. This phenomenon is more pronounced when the FOV is frequently occluded by hand: a large number of ghosting artifacts occurred in the Gated–LMS and ST–LMS, as shown in the blue box in the bottom row of Figure 8b,c. Our algorithm preserved most of the image details, and Figure 8d shows that there were no obvious degradation or ghosting artifacts during the imager jittering and hand occlusion of the FOV. The visual effect of the corrected image processed by our algorithm is satisfactory.

**Figure 9:**
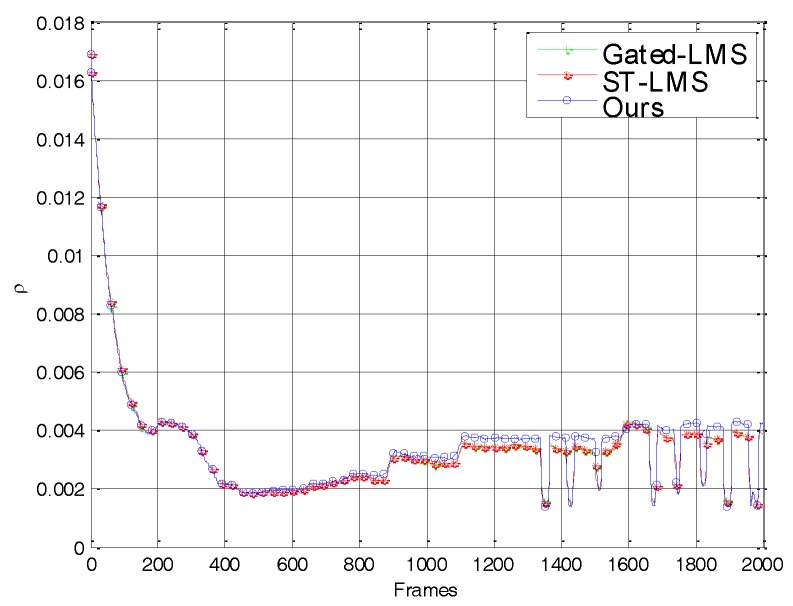
Roughness versus frame number for the tested algorithms using real nonuniformity data.

Figure 9 shows the roughness curves of the image sequences processed by the tested algorithms. The figure shows that the roughness curves drop rapidly in the first 540 frames and that they are almost coincident. This further proves that the adaptive edge detection did not hinder the removal of nonuniformity noise. Then, during the frequent occlusion of the FOV, the roughness curves appear to change in a cyclical manner. When the hand was removed, the image roughness became larger. In this case, the roughness of our algorithm was the highest because ST–LMS and Gated–LMS caused image degradation. The roughness was reduced when the FOV was blocked by hand. The roughness of our algorithm was lowest at this time because both ST–LMS and Gated–LMS resulted in ghosting artifacts. The experiment further shows that the adaptive spatial threshold makes our algorithm have a stronger deghosting ability than ST–LMS and Gated–LMS, while not reducing the denoising ability.

**Figure 10:**
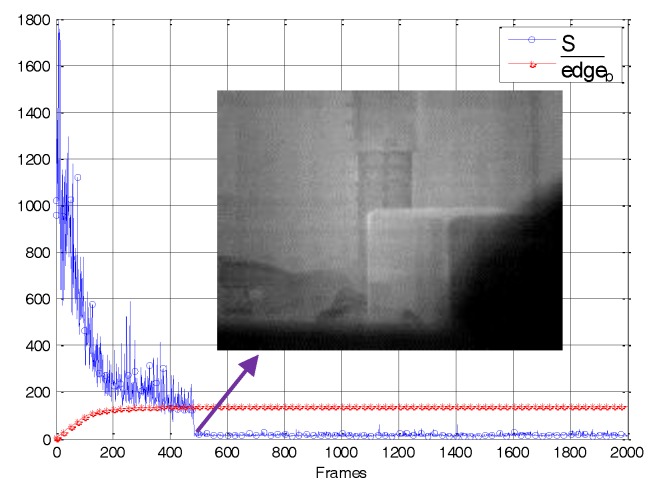
The variation of the important variables in our algorithm during the real nonuniformity data correction.

Figure 10 shows the frame-by-frame variation of the correction matrix gradient $\bar{{\mathrm{edge}}_{b}}$ and the adaptive threshold *S*. It can be seen that the curve of $\bar{{\mathrm{edge}}_{b}}$ gradually rises to a certain point before stabilizing. For 481 frames, while the temporal variation was lower than the threshold, the corrected image noise was very weak, as indicated by the arrow in Figure 10. The *S* almost represented the noise level as evaluated by the subjective visual. After 481 frames, *S* drops sharply, before remaining at around 20. Consequently, the spatial threshold *S* in our algorithm adaptively changed according to the real residual nonuniformity noise.

The second real data was acquired by a 384 × 288 FPA uncooled imager from North Guang Wei [23]. The imager works at 50 FPS and the F-number of the optics is 0.75. The outdoor scene was observed at 9 p.m., and uncorrected raw data with severe FPN was obtained. The imager moved randomly without any intentional jitter and occlusion. In total, there were 3315 frames.

**Figure 11:**
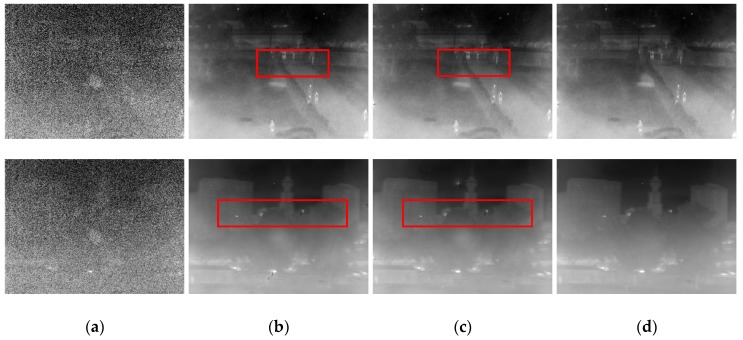
NUC performance comparison when the imager moved randomly without any intentional jitter and occlusion: (**a**) uncorrected image; (**b)** Gated–LMS; (**c**) ST–LMS; (**d**) our image. The top row is the 794th frame. The bottom row is the 3289th frame.

Compared with the previous experiment, the images in this group were noisier. Thus, the ST–LMS used *Ts* = 200. The other parameters in the algorithms were the same as those used in the previous experiment. Figure 11 shows some typical images during the calibration. It shows that the scene-based non-uniformity correction performed well even when the original image was mostly covered by noise. However, the red boxes in Figure 11b,c show that Gated–LMS and ST–LMS also produced some ghosting, even when the imager maintained random motion. In this situation, the performance of our algorithm was still better, as shown in Figure 11d.

## 5. Efficiency Analysis

In order to analyze the efficiency of the algorithm, we calculated the runtime of the algorithms for a single image on Matlab (MathWorks, Natick, MA, USA). The computer’s CPU was Intel Core i7-3540M (3.00GHz). As the conditional statement in the algorithm renders the processing capacity different for each frame, we calculated the average processing time of 100 images, as shown in Table 1.

Obviously, the adaptive edge detection module added in our algorithm increased the computational complexity of the algorithm, rendering its PC time consumption significantly higher than that of the other two algorithms. To analyze the degree of efficiency degradation, we counted all the computational resources per pixel from Equation (13) to Equation (22). The resulting data used by the different algorithms—the comparison, adding/subtracting, multiplying, and dividing—is listed in Table 2. The desired image was obtained by a four neighborhood filter, and the spatial correlation window in ST–LMS was 7 × 7. The *W* equaled 7 in our algorithm.

The table shows that our algorithm consumes more than the double ST–LMS’s consumption in logical operations, addition operations, and multiplication operations. These increased computational costs mainly concern Equations (13) and (20). Fortunately, our algorithm exhibits only one time-consuming division operation in excess of the other two algorithms. It derives from the local variance calculation in Equation (20), which is only used as comparison. Given this, the division can be optimized as a combination of multiplication and shift operations without affecting the result.

## 6. Conclusions

In this paper, we present an improved learning rate rule which combines adaptive threshold edge detection with a temporal gate in the NN-based NUC algorithm. The adaptive edge detection threshold can be automatically adjusted according to the residual nonuniformity of the corrected image, so that the algorithm has the ability to continuously suppress ghosting artifacts without affecting the convergence speed.

The proposed AST–LMS method was compared with Hardie et al.’s Gated–LMS and Fan et al.’s ST–LMS methods in the experiments by using infrared video sequences with simulated nonuniformity noise and real nonuniformity noise, respectively. The simulated experiment showed that when the imager was in stable motion, AST–LMS had an equivalent PSNR to Gated–LMS and ST–LMS. However, AST–LMS had a higher PSNR than ST–LMS and Gated–LMS when the imager was jittering. The real data experiments showed that our algorithm achieves a better deghosting ability while retaining the NUC performance, regardless of whether the imager has an intentional jitter or occlusion. The roughness index of AST–LMS is better than those of Gated–LMS and ST–LMS. These results show that the adaptive method in this paper is a reasonable option for the spatial threshold in the previous deghosting method. In addition, just like the analysis in the simulated experiment, the temporal variation of the correction matrix gradient proposed in the algorithm can be used as a criterion for the convergence of the algorithm.

The nonuniformity correction algorithm always runs in the embedded field-programmable gate arrays circuit of the infrared imager. According to our previous work on the real time nonuniformity correction algorithm [5], the added edge detection and adaptive threshold calculation in this paper will increase the power consumption of the circuit and the design difficulty of the pipeline.

The proposed algorithm divides the nonuniformity correction process into denoising and deghosting stages. However, in the denoising stage, pixels with faint details are still likely to produce “ghosting” because their gradients are similar to nonuniformity noise, and consequently their correction parameter updating is not limited. Future work may focus on using machine learning methods to improve the accuracy of incident radiation estimation.

## Figures and Tables

**Table 1:** Matlab runtime comparison of the algorithms.

Method	Gated–LMS	ST–LMS	Ours
Time(s)	0.0512	0.4627	0.7472

**Table 2:** The comparison of the computational resources per pixel.

	Logical Operation	Adding	Multiplication	Division
Gated–LMS	2	19	8	4
ST–LMS	77	58	8	4
Ours	161	131	19	5

## Supplementary Materials

The following are available online at www.mdpi.com/1424-8220/18/1/211/s1, Video S1: Algorithms performance comparison with simulated nonuniformity. Video S2: Algorithms performance comparison with real nonuniformity. In Videos S1 and S2, the top row are the original image and the image processed by Gated–LMS, from left to right. The bottom row are images processed by ST–LMS and our method, from left to right.
